# Non-Alcoholic Fatty Liver Disease in Patients with Type 2 Diabetes: Evaluation of Hepatic Fibrosis and Steatosis Using Fibroscan

**DOI:** 10.3390/diagnostics10030159

**Published:** 2020-03-14

**Authors:** Tran Thi Khanh Tuong, Dang Khoa Tran, Pham Quang Thien Phu, Tong Nguyen Diem Hong, Thien Chu Dinh, Dinh Toi Chu

**Affiliations:** 1 Department of Internal Medicine, Pham Ngoc Thach University of Medicine, Ho Chi Minh City 700000, Vietnam; drkhanhtuong@gmail.com (T.T.K.T.); phu2981992@gmail.com (P.Q.T.P.); 2 Department of Anatomy, Pham Ngoc Thach University of Medicine (PNTU), Ho Chi Minh City 700000, Vietnam; khoatrandr@gmail.com; 3 Dai Phuoc Polyclinic of Ho Chi Minh, Ho Chi Minh City 700000, Vietnam; diemhong1702@gmail.com; 4 Institute for Research and Development, Duy Tan University, 03 Quang Trung, Danang 550000, Vietnam; 5 Department of Human and Animal Physiology, Faculty of Biology, Hanoi National University of Education, Hanoi 100000, Vietnam

**Keywords:** NAFLD, NASH, diabetes, fibrosis, steatosis, FibroScan, CAP

## Abstract

Patients with type 2 diabetes mellitus (T2DM) are at increased risk of non-alcoholic fatty liver disease (NAFLD) and might eventually progress to advanced fibrosis, cirrhosis and hepatocellular carcinoma (HCC). Recommendations on whether to screen for NAFLD in diabetic patients remains conflicted between major guidelines. Transient elastography using FibroScan with CAP (controlled attenuation parameter) can assess both liver steatosis and fibrosis simultaneously. This paper took a new look at the prevalence of NAFLD and the severity of fibrosis among T2DM patients in Vietnam. The study was conducted using a cross-sectional design in T2DM adults who attended Dai Phuoc Ho Chi Minh Polyclinic and Polyclinic of Pham Ngoc Thach University of Medicine. Liver steatosis and fibrosis was assessed by FibroScan. NAFLD was diagnosed if CAP > 233 dB/m (steatosis > 5%). Data were analyzed using STATA 12 software program. We found that a total of 307 type 2 diabetic patients qualified for the study’s criteria. The prevalence of NAFLD in T2DM patients based on FibroScan was 73.3%. Rates of mild, moderate and severe steatosis were 20.5%, 21.8% and 30.9%, respectively. The prevalence of significant fibrosis (≥ F2), advanced fibrosis (≥ F3) and cirrhosis (F4) was 13.0%, 5.9% and 3.6%, respectively. On multivariate analysis, aspartate aminotransferase (AST) (OR: 1.067; 95% CI: 1.017–1.119; p = 0.008) and platelet levels (OR: 0.985; 95% CI: 0.972–0.999; p = 0.034) were independent of risk factors of advanced fibrosis. Thus, our study supports screening for NAFLD and for evaluating the severity of liver fibrosis in T2DM patients.

## 1. Introduction

Estimated incidences of non-alcoholic fatty liver disease (NAFLD) worldwide have increased twice in the last two decades, while the incidences of other chronic liver diseases (CLD) have remained unchanged or are on downward trends [1]. Traditionally, NAFLD has been reported as a burden condition only in the United States (US) or Western countries. However, nowadays, urbanization has brought about sedentary lifestyles and overnutrition in many Asian countries, leading to the increase of obesity and metabolic disorders. As the result, NAFLD has been recognized as the most common CLD in Asia [2,3]. Currently, the population prevalence of NAFLD in the US and Europe is approximately 30%, whereas in Asia the prevalence is around 25%, like many Western countries [3].

Although almost all NAFLD patients have simple steatosis only, 10%–20% of patients represent the active form: non-alcoholic steatohepatits (NASH) [1]. NASH can develop into liver fibrosis, progressing to cirrhosis, heptatocellular carcinoma (HCC), and finally end-stage liver failure. Moreover, NAFLD patients are at 64 times higher risk of cardiovascular disease (CVD) including coronary artery disease and stroke than patients without NAFLD [4]. Mortality in NAFLD patients is mostly due to CVD events, markedly exceeding the common population [5].

Type 2 diabetes mellitus (T2DM) is the main risk factor of NAFLD. Patients with T2DM are at a greater risk of NAFLD and have a higher rate of death and progression to cirrhosis than non-diabetic individuals [6]. Therefore, screening for NAFLD and evaluating liver fibrosis in the diabetic population is extremely essential for early detection and management, preventing the progression to advanced fibrosis, cirrhosis and HCC.

NAFLD is diagnosed when there is evidence of ≥ 5% hepatic steatosis either by histology or imaging and absence of secondary causes of fatty accumulation [7,8]. FibroScan with controlled attenuation parameter (CAP) measurement can assess hepatic steatosis levels. Nevertheless, transient elastography (TE) performed by FibroScan is recommended by the American Association for the Study of Liver Diseases (AASLD) and European Association for the Study of the Liver (EASL) to evaluate liver fibrosis in patients with NAFLD [7,8]. This is a non-invasive, simple-to-perform imaging modality with high accuracy to assess liver stiffness and hepatic fat deposition. Thus far there has been little knowledge on the prevalence of NAFLD and liver fibrosis in diabetic populations in Vietnam. Therefore, our study proposed to estimate the prevalence of CAP-defined NAFLD and the severity of liver fibrosis by TE performance in T2DM patients.

## 2. Materials and Methods 

### 2.1. Study Design: This Study Was A Cross-Sectional Analysis

### 2.2. Populations

#### 2.2.1. Inclusion Criteria

Patients older than 18 years with known T2DM or previously unknown diabetes but a displaying fasting glucose of ≥ 126 mg/dL (7 mmol/L) or HbA1c ≥ 6.5% [9] and who were admitted for medical check-up at Dai Phuoc Ho Chi Minh Polyclinic and the Polyclinic of Pham Ngoc Thach University of Medicine between 07/2018 and 06/2019 were included in the study. All procedures performed in the studies involving human participants were in accordance with the ethical standards of the local Pham Ngoc Thach University of Medicine’s Ethical Committee (No.05/HDDD), and with the 1964 Helsinki declaration and its later amendments or comparable ethical standards.

#### 2.2.2. Exclusion Criteria

(1) Excessive daily alcohol intake ≥ 30 g in men and ≥ 20 g in women. (2) Causes of secondary hepatic steatosis: pregnancy, abuse of steatogenic drugs (aminodarone, tamoxifen, methotrexate, corticosteroid, estrogen), severe malnutrition. (3) Positive hepatitis B surface antigen or hepatitis C viral antibody. (4) Other chronic liver diseases: autoimmune hepatitis, hemochromatosis, Wilson’s disease, primary biliary cirrhosis, drug-induced hepatitis. (5) Measurement failure or unreliable results on TE (ascites, cholestasis, acute exacerbation or hepatitis flare with ALT > 5 times baseline, interquartile range to median ratio (IQR/med) > 30% or success rate < 60%). 

### 2.3. Sample Size

With a predicted prevalence of NAFLD defined on FibroScan as 72.8% in a previous report [10], our study needed a minimum sample size of 305 patients with T2DM to evaluate the prevalence with a 95% confidence interval and 5% precision.

### 2.4. Performance

#### 2.4.1. Clinical Assessment

Patients who met all study criteria were enrolled. Participating subjects who had given their written informed consent were checked for medical histories, physical examinations and laboratory tests.

#### 2.4.2. FibroScan Examination

Participants were required to fast for at least 3 h before the procedure. TE was performed by a single experienced operator using FibroScan 530 compact (Echosens, Paris, France) with a standard M probe. Liver stiffness measurement (LSM) and CAP were described by the median of 10 successful measurements. LSM was considered reliable only if IQR/med < 30% and success rate > 60%. CAP score was considered reliable if 10 valid acquisitions were obtained.

Significant liver fibrosis (≥F2), advanced fibrosis (≥F3) and cirrhosis (F4) based on TE were defined as LSM ≥ 7 kPa, ≥ 8.7 kPa and ≥ 11.5 kPa, respectively, according to the previous study of LSM using TE in NAFLD patients [11]. Patients were considered to have NAFLD based on FibroScan if CAP > 233 dB/m [12] with mild, moderate, severe steatosis defined as CAP 234–269 dB/m (S1), 270–300 dB/m (S2) and ≥ 301 dB/m (S3), respectively.

Obesity was established as a body mass index (BMI) ≥ 25 kg/m^2^ for adult Asians, as proposed by the World Health Organization (WHO) [13]. Waist circumference ≥ 80 cm in females and ≥ 90 cm in males indicated central obesity for adult Asians [14]. Hypertension was considered if the patient had a history of hypertension and was taking antihypertensive drugs or if their systolic blood pressure (SBP) was ≥ 140 mmHg or diastolic blood pressure (DBP) ≥ 90 mmHg [15]. The presence of dyslipidemia was ascertained in patients who were previously diagnosed and were taking lipid-lowering drugs or who displayed at least 1 of 4 features: cholesterol > 200mg/dL (>5.2 mmol/L), triglyceride > 150 mg/dL (>1.7 mmol/L), HDL-C < 40mg/dL and LDL-C > 130 mg/dL [16]. Patients with metabolic syndrome (MS) were diagnosed following the standard proposed by the International Diabetes Federation (IDF) [14]: central obesity with at least 2 of the following points: triglyceride ≥ 150 mg/dL (≥1.7 mmol/L); HDL-C < 40 mg/dL (<1.06 mmol/L) in males or < 50 mg/dL (<1.3 mmol/L) in females, blood pressure ≥ 130/80 mmHg or on antihypertensive medication with fasting glucose plasma ≥ 100 mg/dL or the presence of T2DM. Alanine aminotransferase (ALT) and aspartate aminotransferase (AST) were determined to be elevated when results were ≥ 33 IU/L in males and ≥ 25 IU/L in females [17]. Patients with NAFLD with increased ALT or at least with significant fibrosis were diagnosed with NASH [18].

### 2.5. Data Analysis

Data were summarized and examined using STATA 12 software. Continuous variables with normal distribution were expressed as mean and standard deviation (SD), whereas median with IQR (25 th–75 th) were used to illustrate results with asymmetric distribution. In addition, categorical elements were represented as numbers and percentages. Comparison between continuous variables was performed by t-test or Mann–Whitney test where appropriate. Chi-square test was performed to deal with the categorical factors. Logistic regression was used to investigate patients’ characteristics independently associated with advanced fibrosis. Variables proven to be significant on univariate analysis were selected for multivariate analysis. *p*-value < 0.05 was assumed to be statistically significant.

## 3. Results

### 3.1. Population Characteristic

From 7/2018 to 6/2019, a total of 311 patients qualified for the study’s inclusions. Four patients were eliminated due to unreliable FibroScan results (*n* = 3) and failed examination (*n* = 1). Data from 165 women and 142 men were analyzed. The average age was 56.8 ± 11.3. The BMI of the sample was normally distributed, with a mean of 24.9 ± 2.9 kg/m^2^. Approximately one-half of the study population had a BMI greater than 25 kg/m^2^. The mean waist circumference was significantly higher (*p* < 0.05) in men (90.4 ± 8.9 cm) compared to women (82 ± 9.1 cm). However, there was no statistical difference in the prevalence of central obesity between men and women, which consisted of 63.5% for both sexes. The patient characteristics are shown in Table 1.

### 3.2. Prevalence of NAFLD in Type 2 Diabetic Patients

**Figure 1:**
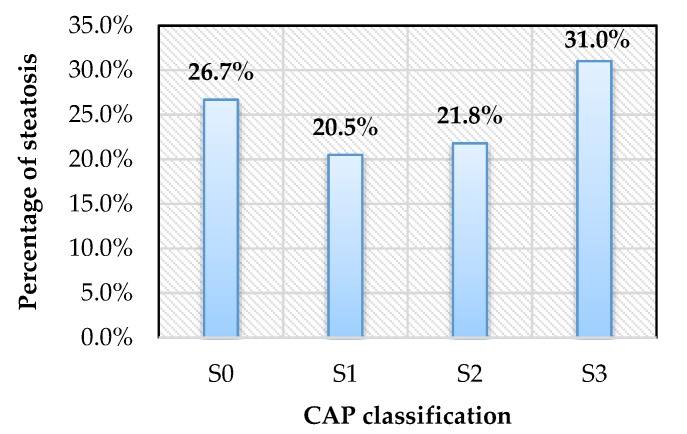
Hepatic steatosis on FibroScan with controlled attenuation parameter (CAP). S0, CAP ≤ 233 dB/m; S1, CAP 234–269 dB/m; S2, CAP 270–300 dB/m; S3, CAP ≥ 301 dB/m.

**Figure 2:**
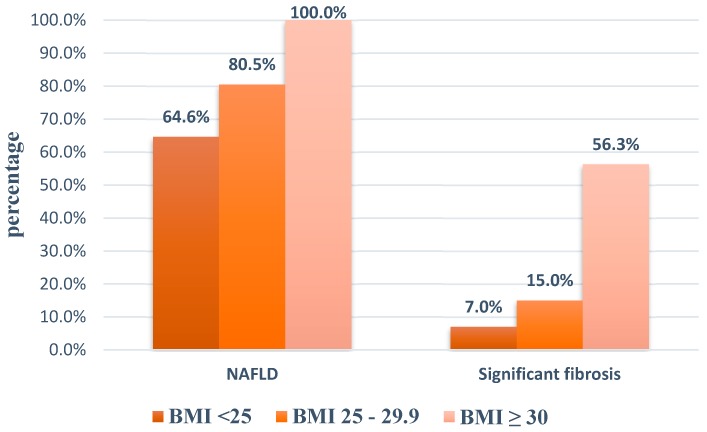
Proportion of patients with NAFLD and significant fibrosis according to BMI. (BMI, body mass index. NAFLD, non-alcoholic fatty liver disease).

Among 307 patients having reliable results, the prevalence of NAFLD based on TE was 73.3%, with mild, moderate and severe hepatic steatosis consisting of 20.5%, 21.8% and 31.0%, respectively (Figure 1). Moreover, the prevalence of NAFLD increased with the increasing BMI (Figure 2).

T2DM patients with NAFLD had greater BMI and waist circumference than patients without NAFLD, and tended to be obese and centrally obese. As far as we know, these patients also more easily suffered from metabolic disorders such as hypertension, dyslipidemia or metabolic syndrome. Higher levels of ALT, AST and gamma-glutamyltransferase (GGT) were also found in NAFLD patients. 

### 3.3. Prevalence of Hepatic Fibrosis Stages in Type 2 Diabetic Patients 

The proportion of at least significant fibrosis, advanced fibrosis and cirrhosis was 13%, 5.9% and 3.6%. Significant fibrosis was found to be increased with increasing BMI (Figure 2). Patients with advanced fibrosis were more likely to be centrally obese as they had greater BMI and waist circumference and probably sustained MS. AST, ALT and GGT were higher and platelet levels were lower in individuals with advanced fibrosis (Table 2). 

FibroScan revealed that hepatic fibrosis was mostly found in patients with NAFLD. Majority of patients with at least significant fibrosis (≥F2) had NAFLD (38/40) while all of patients with advanced fibrosis (≥F3) and cirrhosis were identified with NAFLD based on FibroScan.

### 3.4. NASH in Diabetic Patients

Forty-eight-point five percent of diabetic patients were identified with NASH after excluding other causes of CLD. ALT was observed to be elevated in a great number of those patients (93.3%) while 10 individuals had normal ALT but significant fibrosis (≥F2) on FibroScan (6.7%).

### 3.5. Factors Predicting Advanced Fibrosis in Type 2 Diabetic Patients

It can be seen in Table 3 that on univariate analysis, BMI, waist circumference, ALT, AST, GGT, platelet and the presence of central obesity were significantly related to advanced fibrosis in T2DM patients. However, on multivariate analysis, independent points associated with advanced fibrosis were AST (OR: 1.067; 95%CI: 1.017–1.119; *p* = 0.008) and platelets (OR: 0.985; 95% CI: 0.972–0.999; *p* = 0.034) 

## 4. Discussion

In our study of 307 T2DM patients who underwent TE, we found a high prevalence of NAFLD of 73.3%, including more than one-third of severe steatosis. The proportion of significant fibrosis, advanced fibrosis and cirrhosis was 13.0%; 5.9% and 3.6%, respectively. Forty-eight-point five percent of patients in our study had NASH, which consisted of more than 65% NAFLD patients. AST and platelets were independent predictive factors of advanced fibrosis.

We compared our results with other prior studies. The studies of Kwok (Hongkong) and Lee-Lee Lai (Malaysia) showed that the prevalence of NAFLD in diabetic patients based on FibroScan was 72.8% and 72.4% with the CAP cut-offs of 222 dB/m and 263 dB/m, respectively [10,19]. Overall, our findings were in line with the previous studies despite different CAP cut-offs. The cut-off in ours was 233 dB/m, which was the cut-off being using at our center as well. Our study populations had considerably lower mean BMI, that is, 24.9 kg/m^2^ versus 28.2 kg/m^2^ and 26.6 kg/m^2^ in the study by Kwok and Lee-Lee Lai, respectively. BMI is known to impact CAP measurements [20]. Furthermore, more recent evidence highlights that the epidemiology of NAFLD is significantly influenced by ethnicity [21,22], which may also take a crucial part in designing screening programs of individual countries.

Regarding the percentage of diabetic patients with NAFLD, the range is between 49%–69.4% [6]. We believe that our data is in agreement with the published literature. There are various reported prevalence rates of NAFLD in diabetes depending on different methods used to evaluate the liver. Liver biopsy is considered the “gold standard” for both steatosis and fibrosis assessment. However, this is an invasive procedure with rising risk of complications. Abdominal ultrasonography was used to detect hepatic steatosis by most of the previous research, which is an inexpensive and widely available equipment. However, liver ultrasound may be inadequately accurate if there is less than 33% hepatic fat accumulation [23]. Recently, FibroScan with CAP measurement is a novel non-invasive, easy-to-perform tool developed to assess both hepatic steatosis and fibrosis simultaneously with high sensitivity and specificity [24]. Screening for NAFLD in T2DM individuals using FibroScan/CAP is recommended in the latest Asia–Pacific Working Party on Non–Alcoholic Liver Disease guidelines [25]. Moreover, we also found increasing rates of MS, hypertension, dyslipidemia and obesity in patients with NAFLD. Therefore, it is important to evaluate all components of MS in NAFLD patients and vice versa, and to screen for NAFLD in patients with obesity, MS, hypertension or dyslipidemia.

FibroScan in our center is not equipped with an XL probe, so the accuracy of LSM in individuals having a BMI greater than 28 kg/m^2^ may have been influenced [26]. As far as we know, there are two other studies using only an M probe and the same cut-offs for fibrosis stages like ours. In their study, Sobhonslidsuk and colleagues pointed out that the prevalence of significant fibrosis and advanced fibrosis based on TE was 16.1% and 5.8%, respectively [27]. Apparently, these findings concurred well with our study findings. This is likely explained by the similarities in population characteristics between Vietnamese and Thai studies. Otherwise, the study of de Lédingen and colleagues discovered that patients with advanced fibrosis accounted for 12.9% [28], which was remarkably higher compared to our results. This may be because of the diversity in patient characteristics between two studies, for instance, BMI and waist circumference in the French study (27.2 kg/m^2^ and 97.2 cm) was greater than in ours (24.9 kg/m^2^ and 85.9 cm). Another possible reason for this is that liver fibrosis related to NAFLD in Asia is not as common as in Europe. Natural development of NAFLD to end-stage liver disease usually progresses slowly, lasting for around 10 to 20 years, and the latter appearance of NAFLD results in a lower proportion of advanced fibrosis or cirrhosis in many Asian surveys compared to those in Europe [3].

The presence of NAFLD in T2DM patients can lead to substantial risk of liver fibrosis. In our study, the proportion of fibrosis severities in patients with NAFLD were considerably higher than patients without NAFLD (*p* < 0.05). Vice versa, diabetes is associated with increased risk of advanced fibrosis as well. Another study using FibroScan to evaluate liver fibrosis in both diabetic and non-diabetic patients revealed that advanced fibrosis in diabetic subjects was 26.9% while only 4.5% of non-diabetic individuals had advanced fibrosis [29]. In addition, it is important to note that among diabetic patients with BMI < 25 kg/m^2^, 64.6% patients still had NAFLD and 7% had significant fibrosis. In previous studies about NAFLD, such non-obese patients usually have other components of MS [30]. Obviously, the presence of diabetes itself is one of the components of MS and the prevalence of MS was high among NAFLD patients in our study as well. These non-obese populations still face metabolic problems and the risk of CVD equivalently to obese individuals [31].

NAFLD is believed to be the leading reason of ALT elevation in type 2 diabetic patients [6]. Our study revealed that elevated liver enzymes in patients with NAFLD were mainly due to increased ALT related to NASH after dropping out other causes of CLD. Ninety percent of NASH patients had increased ALT and AST, but in 10% of NASH patients the ALT may be normal [1]. We figured out that nearly 7% of patients had significant fibrosis but not elevated ALT. In our findings, AST and platelet levels appeared to be independent predictive factors of advanced fibrosis in T2DM patients. ALT is mainly located in the cytoplasm of the hepatic cell. By contrast, AST is mostly present in the mitochondrial membrane (80%), while the remainder is in the cytosol [32]. Chronic liver injuries can damage the mitochondria leading to the increasing release of AST into the blood, which is commonly seen in patients with advanced fibrosis. Moreover, hepatic fibrosis progression can also result in reduced elimination of AST in hepatic sinusoid contributing to the elevation of AST serum [33]. On the other hand, advanced fibrosis can precede portal hypertension inducing thrombocytopenia due to increased sequestration and destruction of platelets in the enlarged spleen [34]. Lower platelet levels predict a more extreme hepatic fibrosis. 

Our work has several limitations. First of all, we do not have an XL probe to perform FibroScan examination on patients with BMI greater than 28 kg/m^2^. Secondly, owning to lack of liver biopsy for diagnostic confirmation of TE findings, we do not have the precise prevalence of NASH. Finally, our findings may not be generalizable to patients with T2DM in all primary care settings as well as diabetic inpatients at hospitals. Future studies should concentrate on enhancing the quality of the current topic.

## 5. Conclusions

To sum up, we found that the prevalence of NAFLD based on transient electrography is high among T2DM patients. Patients with NAFLD are at an increased risk of advanced fibrosis than those without NAFLD. Our study supports screening for NAFLD and evaluating severity of hepatic fibrosis in T2DM patients.

## Figures and Tables

**Table 1:** Features of patients with and without non-alcoholic fatty liver disease (NAFLD) based on transient elastography.

	Patients with NAFLD (*n* = 225)	Patients without NAFLD (*n* = 82)	*p*
Age (year)	56.5 ± 10.5	57.6 ± 13.1	0.508
Male (%)	46.7	45.1	0.810
Duration of DM (year)	3	3	0.439
SBP (mmHg)	125.2 ± 13.9	122.7 ± 14.0	0.164
DBP (mmHg)	77.7 ± 9.0	74.7 ± 9.3	**0.010**
Hypertension (%)	65.8	52.4	**0.033**
BMI (kg/m^2^)	25.4 ± 2.8	23.4 ± 2.9	**<0.001**
Waist circumfenrence (cm)	87.9 ± 9.3	80.5 ± 9.4	**<0.001**
Obesity (%)	54.7	31.7	**<0.001**
Central obesity (%)	72.4	39.0	**<0.001**
Dyslipidemia (%)	93.8	73.2	**<0.001**
MS (%)	68.9	36.6	**<0.001**
Glucose (mg/dL)	136 (120–168)	125 (112–153)	**0.016**
Cholesterol (mg/dL)	186.7 ± 52.1	173.5 ± 51.6	0.050
Triglyceride (mg/dL)	199.1 (136.4–264)	127.3 (87–193.2)	**<0.001**
HDL (mg/dL)	50.6 ± 13.0	56.6 ± 15.0	**0.002**
LDL (mg/dL)	114.2 ± 39.0	100.3 ± 41.5	**0.009**
ALT (U/L)	34 (23.4–54.1)	20.8 (15.9–29.8)	**<0.001**
AST (U/L)	24.4 (20.1–33.9)	20.2 (15.8–23.7)	**<0.001**
GGT (U/L)	46 (36.7–77.6)	37.5 (21.4–46.8)	**<0.001**
Platelet (K/μL)	224 ± 53	229 ± 61	0.538

The diagnosis of NAFLD was based on CAP ≥ 233 dB/m. DM, diabetes mellitus; SBP, systolic blood pressure; DBP, diastolic blood pressure. BMI, body mass index. MS, metabolic syndrome. HDL, high density lipoprotein. LDL, low density lipoprotein. *p*-values were determined by t-test, and Mann-Whitney test or Chi-square test, where appropriate.

**Table 2:** Features of patients with and without advanced fibrosis on transient elastography.

	Patients with Advanced Fibrosis (*n* = 18)	Patients without Advanced Fibrosis (*n* = 289)	*p*
Age (year)	58.7 ± 11.3	56.7 ± 11.2	0.725
Male (%)	38.9	46.7	0.518
Duration of DM (year)	6.5 (3–10)	3 (2–6)	0.056
SBP (mmHg)	123.3 ± 12.6	124.6 ± 14.0	0.690
DBP (mmHg)	76.7 ± 8.6	76.9 ± 9.2	0.920
Hypertension (%)	72.2	61.6	0.367
BMI (kg/m^2^)	26.3 ± 3.1	24.8 ± 2.9	**0.039**
Waist circumference (cm)	90.7 ± 9.0	85.6 ± 9.9	**0.034**
Dyslipidemia (%)	94.4	87.9	0.402
Obesity (%)	66.7	47.4	0.113
Central obesity (%)	88.9	61.9	**0.021**
MS (%)	88.9	58.5	**0.011**
Glucose (mg/dL)	147.5 (132–170)	133 (117–165)	0.113
Cholesterol (mg/dL)	174.5 ± 50.2	183.7 ± 52.4	0.469
Triglyceride (mg/dL)	177.1(142.3–244.9)	182.2 (109.9–250.7)	0.547
HDL–C (mg/dL)	48.9 ± 11.9	52.4 ± 13.9	0.248
LDL–C (mg/dL)	105.2 ± 41.2	110.8 ± 40.1	0.582
ALT (U/L)	60.6 (40.8–80.6)	29.7 (19.5–42.2)	**<0.001**
AST (U/L)	45.3 (33.2–67.9)	22.6 (18.2–29.2)	**<0.001**
GGT (U/L)	87.4 (62.4–173.9)	46 (30.4–63.4)	**<0.001**
Platelet (K/μL)	186 ± 41	228 ± 55	**0.005**

The diagnosis of advanced fibrosis was based on LSM ≥ 8.7 kPa. DM, diabetes mellitus; SBP, systolic blood pressure; DBP, diastolic blood pressure; BMI, body mass index; MS, metabolic syndrome; HDL, high density lipoprotein; LDL, low density lipoprotein; p values were determined by t-test, Mann-Whitney test or Chi-square test, where appropriate.

**Table 3:** Associations between the presence of advanced fibrosis on transient elastography and T2DM patients’ characteristics by univariate and multivariate logistic regression analyses.

	Univariate Analysis			Multivariate Analysis		
	OR	95% CI	p	Adjusted OR	95% CI	*p*
Age	1.016	0.974–1.061	0.454			
Gender	0.726	0.273–1.925	0.520			
Duration of DM	1.092	1.000–1.193	0.050			
BMI	1.191	1.006–1.411	**0.043**	1.152	0.837–1.586	0.385
Obesity	2.219	0.811–6.073	0.121			
Waist circumference	1.055	1.004–1.110	**0.036**	0.964	0.870–1.069	0.486
Central obesity	4.916	1.109–21.792	**0.036**	3.220	0.460–22.527	0.239
SBP	0.993	0.959–1.029	0.713			
DBP	0.997	0.947–1.051	0.924			
Hypertension	1.621	0.563–4.672	0.371			
Cholesterol	0.997	0.987–1.006	0.485			
HDL	0.981	0.945–1.019	0.322			
LDL	0.997	0.984–1.009	0.583			
Triglyceride	0.999	0.996–1.003	0.771			
Glucose	1.003	0.995–1.011	0.429			
ALT	1.022	1.009–1.035	**0.001**	0.983	0.954–1.013	0.271
AST	1.055	1.032–1.077	**<0.001**	1.067	1.017–1.119	**0.008**
GGT	1.007	1.003–1.010	**<0.001**	0.999	0.995–1.005	0.933
Platelet	0.981	0.970–0.993	**0.002**	0.985	0.972–0.999	**0.034**

DM, diabetes mellitus; SBP, systolic blood pressure; DBP, diastolic blood pressure; BMI, body mass index. HDL, high density lipoprotein; LDL, low density lipoprotein; CI, confidence interval; OR, odd ratio.

## References

[1] LaBrecque D., Abass Z., Anania F., Ferenci P. (2012). Nonalcoholic fatty liver disease and nonalcoholic steatohepatitis. World Gastroenterology Organisation Global Guidelines.

[2] Ashtari S., Pourhoseingholi M., Zali M. (2015). Non-alcohol fatty liver disease in Asia: Prevention and planning. World J. Hepatol..

[3] Jian-Gao F., Seung-Up K., Vincent W. (2017). New trends on obesity and NAFLD in Asia. J. Hepatol..

[4] Targher G., Byrne C., Lonardo A., Zoppini G., Barbui C. (2016). Non-alcoholic fatty liver disease and risk of incident cardiovascular disease: A meta-analysis. J. Hepatol..

[5] Ekstedt M., Franzen L., Mathiesen U., Thorelius L. (2006). Long-term follow-up of patients with NAFLD and elevated liver enzymes. Hepatology.

[6] Younossi Z., Gramlich T., Matteoni C., Boparai N., Mccullough A. (2004). Nonalcoholic fatty liver disease in patients with type 2 diabetes. Clin. Gastroenterol. Hepatol..

[7] Chalasani N., Younossi Z., Lavine J., Charlton M., Cusi K., Rinella M., Harrison S., Brunt E.M., Sanyal A.J. (2018). The diagnosis and management of nonalcoholic fatty liver disease: Practice guidance from the American Association for the study of liver diseases. Hepatology.

[8] EASL, EASD, EASO (2016). EASL–EASD–EASO clinical practice guidelines for the management of non-alcoholic fatty liver disease. J. Hepatol..

[9] Association A.D., Riddle M. (2018). Classification and diagnosis of diabetes: Standards of medical care in diabetes 2018. Standards of Medical Care in Diabetes—2018.

[10] Kwok R., Choi K., Wong G., Zhang Y. (2015). Screening diabetic patients for non-alcoholic fatty liver disease with controlled attenuation parameter and liver stiffness measurements: A prospective cohort study. Gut.

[11] Wong V., Vergniol J., Wong G., Foucher J. (2010). Diagnosis of fibrosis and cirrhosis using liver stiffness measurement in nonalcoholic fatty liver disease. Hepatology.

[12] Karlas T., Petroff D., Garnov N., Bohm S. (2014). Non-invasive assessment of hepatic steatosis in patients with NAFLD using controlled attenuation parameter and H-MR spectroscopy. PLoS ONE.

[13] World Health Organization (2000). The Asia-Pacific Perspective: Redefining Obesity and Its Treatment.

[14] International Diabetes Federation (2006). The IDF Consensus Worldwide Definition of the Metabolic Syndrome.

[15] Williams B., Mancia G., Spiering W., Agabiti Rosei E., Azizi M., Burnier M., Clement D.L., Coca A., de Simone G., Dominiczak A. (2018). 2018 ESC/ESH guidelines for the management of arterial hypertension. Eur. Heart J..

[16] National C.E.P.C.C. (2002). National Cholesterol Education Program Expert Panel on Detection, Evaluation, and Treatment of High Blood Cholesterol in Adults (Adult Treatment Panel III).

[17] Kwo P., Cohen S., Lim J. (2016). ACG practice guideline: Evaluation of abnormal liver chemistries. Am. J. Gastroenterol..

[18] Adams L., Feldstein A. (2011). Non-invasive diagnosis of nonalcoholic fatty liver and nonalcoholic steatohepatitis. J. Dig. Dis..

[19] Lai L., Yusoff W., Vethakkan S., Mustapha N. (2019). Screening for non-alcoholic fatty liver disease in patients with type 2 diabetes mellitus using transient elastography. J. Hepatol..

[20] Wang Y., Fan Q., Wang T., Wen J., Wang H., Zhang T. (2015). Controlled attenuation parameter for assessment of hepatic steatosis grades: A diagnostic meta-analysis. Int. J. Clin. Exp. Med..

[21] Bambha K., Belt P., Abraham M., Wilson L.A. (2012). Ethnicity and nonalcoholic fatty liver disease. Hepatology.

[22] Rich N., Oji S., Mufti A., Browning J.D., Parikh N.D., Odewole M., Mayo H., Singal A.G. (2018). Racial and ethnic disparities in nonalcoholic fatty liver disease prevalence, severity, and outcomes in the united states: A systematic review and meta-analysis. Clin. Gastroenterol. Hepatol..

[23] Ryan C., Johnson L., Germin B., Marcos A. (2002). One hundred consecutive hepatic biopsies in the workup of living donors for right lobe liver transplantation. Liver Transplant..

[24] Je´rome B., Paul C. (2012). Controlled attenuation parameter (CAP): A new device for fast evaluation of liver fat?. Liver Int..

[25] Wong V., Chan W., Chitturi S.Y.C. (2018). Asia-Pacifc working party on non-alcoholic fatty liver disease guidelines 2017-Part 1: Definition, risk factors and assessment. J. Gastroenterol. Hepatol..

[26] Wong G., Wong V., Chim A., Yiu K. (2011). Factors associated with unreliable liver stiffness measurement and its failure with transient elastography in the Chinese population. Hepatology.

[27] Sobhonslidsuk A., Pulsombat A., Kaewdoung P., Petraksa S. (2015). Non-alcoholic fatty liver disease (nafld) and significant hepatic fibrosis defined by non-invasive assessment in patients with type 2 diabetes. Asian Pac. J. Cancer Prev..

[28] Lédinghen V., Vergniol J., Gonzalez C., Foucher J. (2012). Screening for liver fibrosis by using FibroScan® and FibroTest in patients with diabetes. Dig. Liver Dis..

[29] Hajiani E., Alavinejad P., Hashemi S., Masjedizadeh A. (2014). Comparison of the transient elastography (fibroscan) results among diabetic and non-diabetic patients with non- alcoholic fatty liver disease. Gastroenterol. Hepatol..

[30] Das K., Das K., Mukherjee P., Ghosh A., Ghosh S. (2010). Nonobese population in a developing country has a high prevalence of nonalcoholic fatty liver and significant liver disease. Hepatology.

[31] Sookoian S., Pirola C. (2017). Systematic review with meta-analysis: Risk factors for non-alcoholic fatty liver disease suggest a shared altered metabolic and cardiovascular profile between lean and obese patients. Aliment. Pharm..

[32] Rej R. (1978). Aspartate aminotransferase activity and isoenzyme proportions in human liver tissues. Clin. Chem..

[33] Wai C., Marrero J.A., Conjeevaram H.S., Lok A.S., Greenson J.K., Fontana R.J., Kalbfleisch J.D. (2003). A simple noninvasive index can predict both significant fibrosis and cirrhosis in patients with chronic hepatitis C. Hepatology.

[34] Witters P., Freson K., Verslype C., Peerlinck K. (2008). Review article: Blood platelet number and function in chronic liver disease and cirrhosis. Aliment. Pharm. Ther..

